# 3D Printing Processability of a Thermally Conductive Compound Based on Carbon Nanofiller-Modified Thermoplastic Polyamide 12

**DOI:** 10.3390/polym14030470

**Published:** 2022-01-25

**Authors:** Zhenxue Zhang, Eleni Gkartzou, Simon Jestin, Dionisis Semitekolos, Panagiotis-Nektarios Pappas, Xiaoying Li, Anna Karatza, Panagiotis Zouboulis, Aikaterini-Flora Trompeta, Nikolaos Koutroumanis, Costas Galiotis, Costas Charitidis, Hanshan Dong

**Affiliations:** 1 School of Metallurgy and Materials, University of Birmingham, Birmingham B15 2TT, UK; x.li.1@bham.ac.uk (X.L.); h.dong.20@bham.ac.uk (H.D.); 2 BioG3D P.C., 1 Lavriou Str., Technological & Cultural Park of Lavrion, 19500 Lavrion, Greece; egkartzou@biog3d.gr (E.G.); akaratza@biog3d.gr (A.K.); pzouboulis@biog3d.gr (P.Z.); 3 CANOE, Le Centre Technologique Nouvelle Aquitaine Composites & Matériaux Avancés, Bât CHEMINNOV–ENSCBP, 33600 Pessac, France; jestin@plateforme-canoe.com; 4 Research Lab of Advanced, Composites, Nanomaterials and Nanotechnology (R-NanoLab), School of Chemical Engineering, National Technical University of Athens, 9 Heroon Polytechniou Str., Zographos, 15780 Athens, Greece; diosemi@chemeng.ntua.gr (D.S.); ktrompeta@chemeng.ntua.gr (A.-F.T.); charitidis@chemeng.ntua.gr (C.C.); 5 FORTH/Institute of Chemical Engineering and High Temperature Chemical Processes, Stadiou Str., Rion, 26504 Patras, Greece; ppappas@iceht.forth.gr (P.-N.P.); nickkoutrou@iceht.forth.gr (N.K.); c.galiotis@iceht.forth.gr (C.G.)

**Keywords:** polyamide 12, carbon nanofiller, carbon nanotube, graphene nanoplatelet, 3D printing, thermal conductivity, compound

## Abstract

A polyamide (PA) 12-based thermoplastic composite was modified with carbon nanotubes (CNTs), CNTs grafted onto chopped carbon fibers (CFs), and graphene nanoplatelets (GNPs) with CNTs to improve its thermal conductivity for application as a heat sink in electronic components. The carbon-based nanofillers were examined by SEM and Raman. The laser flash method was used to measure the thermal diffusivity in order to calculate the thermal conductivity. Electrical conductivity measurements were made using a Keithley 6517B electrometer in the 2-point mode. The composite structure was examined by SEM and micro-CT. PA12 with 15 wt% of GNPs and 1 wt% CNTs demonstrated the highest thermal conductivity, and its processability was investigated, utilizing sequential interdependence tests to evaluate the composite material behavior during fused filament fabrication (FFF) 3D printing processing. Through this assessment, selected printing parameters were investigated to determine the optimum parametric combination and processability window for the composite material, revealing that the selected composition meets the necessary criteria to be processable with FFF.

## 1. Introduction

Conventional materials used for heat transfer applications, such as copper and aluminium, have the advantage of higher thermal conductivity but with higher cost and considerable weight. Low-cost thermoplastic composites with ease of manufacturability and recyclability are alternatives for such applications, although with lower thermal conductivity in comparison to the metallic materials [1,2]. The conduction of thermal energy in a polymer is achieved via a phonon transfer process rather than through vibration in the pure crystalline materials. However, the vibrational frequency mismatches at the interfaces between impurities and lattice defects cause significant phonon “scattering” in polymer materials, which leads to the low thermal conductivities [2]. To enhance the thermal conductivity of polymers, incorporation of highly thermally conductive nanofillers in the host matrix by forming conduction paths is frequently selected as a cost-efficient bulk modification strategy to increase its thermal management applications, including in heat sinks and other components for electronics. The lightweight polymer nanocomposites offer highly specific properties that make them preferable for a wide range of applications. The 0.1 wt% CNTs reinforced polyamide 12 (PA12) nanocomposites prepared by laser sintering have an average 14.2% increase in thermal conductivity compared to that of neat PA12 at temperatures between 100 °C and 175 °C [3]. 

Recent advances in multi-material 3D printing present a potential extension of the product design beyond complex geometries, while functional, single-step manufacturing processes can be exploited by utilizing custom composite materials suitable for multifactorial applications. The fused filament fabrication (FFF) technique is of low cost with a large range of feedstock materials suitable for a wide range of 3D printing applications [4,5]. Thermoplastic materials, such as polylactic acid (PLA) and polyamide (PA) [6], can be melted and extruded as a strand through a nozzle in a line by line and layer by layer manner in FFF, and the extruded material (thermoplastic strands) rapidly solidifies and adheres with the surrounding material, which is especially useful for complex design and advanced functionality of smart products [4,7,8]. The utilization of micro/nanocomposites with multi-material FFF printing, combined with metamaterials and lattice structures, can lead to innovative functional parts for thermal management [9]. Different conductive filaments, carbon-based or metallic reinforcement, to reinforce composites have been utilized for shape morphing applications [10,11] and heat dissipation [12]. By utilizing composite filaments with 1D or 2D (nano) fillers through the FFF 3D printing process, the development of orthotropic components that improve the control of thermal conduction and heat management can be achieved. In this way, thermal pathways or barriers can be incorporated into the 3D printed part through material composition and material path deposition pattern from the FFF tool head [12]. Composite FFF materials with thermally conductive properties are also investigated for high-performance aerospace applications, in consideration of reduced manufacturing cost and weight and ease of fabrication. The influence of filler content on thermal conductivity is significant, indicating that a more thermally conductive filler leads to better thermal conductivity. However, the thermal conductivities of polymer/CNT nanocomposites are relatively low compared with expectations from the intrinsic thermal conductivity of CNTs. The challenge primarily comes from the large interfacial thermal resistance between the CNT and the surrounding polymer matrix, which hinders the transfer of phonon-dominating heat conduction in polymer and CNT [13]. Due to its excellent electrical and thermal conductivity, graphite platelets (GPs)/graphite nanoplatelets (GNPs) were also used to increase the electrical conductivity and mechanical properties of PA12 via selective laser sintering (SLS) [14]. However, the addition of graphite lowered the flowability and the mechanical properties of the PA12 composites compared to the CF-reinforced counterparts [15]. Guo [16] found that larger graphite flakes improved the electrical behavior of the composites to a greater extent than powdered graphite. Although the enhancement of composite performances is not always achieved, it is generally recognized that the synergistic effect can be achieved from the formation of percolative network structures within the polymer due to the presence of different conducting fillers with different aspect ratios and geometrical morphology [17]. Nano- and microfillers, such as carbon black, multiwalled CNTs, or GPs/GNPs, have been used together with CFs to improve the mechanical properties and thermal and electrical conductivities of thermal plastic composites [15].

To produce a printable filament for FFF technology with enhanced functionality, the filler content must also be adjusted according to rheological constraints imposed by the 3D printing technology [18]. In this context, an upper limit in terms of additive amount must be evaluated based on the application requirements, on the shape and size of fillers, and on matrix material properties [19]. A high dispersion and distribution of the selected fillers, together with a low porosity of the final composite filament, are crucial for the improvement of mechanical and thermal properties of the produced material. Meanwhile, it is of high importance to assess FFF processability, since the inclusions alter melting and solidification dynamics through material viscosity alteration and thermal profile of the printed material [10]. Thermoplastic materials present differences in their viscosity and ease of flow during thermal processing, exhibiting a non-Newtonian shear thinning behavior when higher shear rates are applied and non-linear viscosity changes depending on processing temperature. There is plenty of research on the influence of the processing parameters (energy density/filling temperature, filling rate, filling pattern, layer thickness, infill percentage, nozzle size, and manufacturing orientation) on the geometrical accuracy (quality of the surface texture, roundness, and waviness), density, and mechanical properties in additive manufacturing plastic parts based on PLA or PA [20,21,22,23]. FFF process parameters are defined by the user in computer-aided manufacturing (CAM) software and involve a wide variety of toolpath and material processing parameters. However, in the majority of commercial FFF systems, open loop control of the process requires an experimental comparison of actual/nominal characteristics of the thermoplastic strands and compensation factors for each [24]. Taking into account melt viscosity dependence on temperature, shear rate, and pressure conditions, key FFF process parameters that directly affect material flow behavior are printing temperature, printing speed, and nozzle diameter, for a set combination of thermoplastic strand width and height [25]. These parameters define the shear rates and pressure drop imposed on the melt during extrusion and should be kept within a certain processing window for each material system in order to ensure consistent flow and intra-layer coalescence [26]. The printing temperature has also been identified as a main parameter which influences interlayer bonding, structural integrity, and fatigue lifetime of FFF specimens [21]. As heat transfer plays a particular role in determining the temperature history of the merging thermoplastic strands, the subsequent formation of bonds among individual strands arises from complex heat and mass transfer phenomena, coupled with thermal and mechanical stress accumulation and phase changes [27,28]. In this respect, material viscosity, melt strength, storage/loss modulus, melt temperature sensitivity, and phase transitions induced from temperature variation play a significant role in the final part structural integrity and dimensional accuracy. Furthermore, thermoplastic strand height (equal to layer height) and strand width are user-defined parameters that are subsequently employed by the CAM software for the automatic calculation of the filament feed rate; thus, an increase of these values is translated into a nominal increase in material volumetric flow rate to achieve the targeted strand volume [29]. However, material flow imposes physical limitations on the actual strand geometry that can be obtained, leading to over- or under-extrusion when the nominal volumetric flow rate is not in accordance with the actual volumetric flow rate [25]. Although GNPs and CNTs have been widely used to increase the electrical conductivity of PA12, there is hardly any report on improving their thermal conductivity nor the processability of GNP- and CNT-modified PA12 utilizing FFF.

Thermoplastic PA12 has a broad range of applications, such as cable covering and insulating material in the electronics field. In this paper, different nanofillers, in the forms of CNTs, CNT-modified CFs, and GNPs together with CNTs, were used to modify the thermoplastic PA12, and the fillers and the composite were characterized for improved structure and enhanced thermal conductivity. Finally, the processability for 3D printing (FFF) of a printable filament with the highest thermal conductivity, PA12_15%GNP+1%CNT, was assessed, and the processing parameters such as extrusion temperature, printing speed, strand height/width, extrusion multiplier, and retraction distance were optimized for a potential application in a heat sink.

## 2. Materials and Methods

### 2.1. The Materials

The CNT-based materials, either in powder form or grafted on CFs, were synthesized through the chemical vapor deposition (CVD) method. For the first case, the well-established supported catalyst method was utilized, with Fe/zeolite catalyst and acetylene as carbon source [30]. On the contrary, in order to achieve a good grafting of CNTs onto the CFs, different approaches were investigated. Following the supported catalyst approach, the fibers (5–6 mm long) were impregnated in the catalyst solution (Fe particles embedded on zeolite, the same as the conventional procedure for CNT synthesis in powder form) and then left to dry. The coated fibers were spread on an inert substrate and inserted into the CVD reactor. Conventional experimental conditions were set (T = 700 °C, flow rate of acetylene: 60 mL/min, flow rate of Ar: ~200 mL/min). According to the floating catalyst approach, both the ferrocene catalyst and the ethanol precursor were introduced simultaneously into the CVD system, similar to the configuration in [30]. For each batch, 20 g of chopped CFs was introduced into the center of the metallic tube of the CVD reactor. The furnace was heated up to 600 °C in atmospheric air to remove the polymeric sizing on the CFs and to induce mild oxidation. Then, Ar was introduced into the system, which was sealed for 20 min to enable purging. The temperature was raised to 750 °C [30], then the mixture of ethanol and ferrocene (1 g C_10_H_10_Fe diluted in 100 mL C_2_H_5_OH) was boiled, and the vapor was transferred with the carrier gas (flow rate of Ar: ~300 mL/min) in the reactor. The reaction lasted for 2 h.

Graphene nanoplatelets (GNPs) were produced via wet shear exfoliation using graphite obtained from NGS Naturgraphit GmbH, Germany as raw material. The graphite particles exhibited an average lateral size of 500 μm. The appropriate quantity of graphite was dissolved by manual stirring in an aqueous-based solution, and the mixture was transferred into the shear mixing device (Silverson) for the required high-shear stress field to be applied by a 4-blade rotor placed within a fixed screen (stator). At the initiation stage of the shear exfoliation process, the mixer head is driven towards the liquid solution into the vessel and operates at low rotational speed. The speed is gradually increased until the desired predetermined level is reached. The system runs at high speed throughout the remaining mixing time. The GNPs are obtained after drying under controlled temperature. The rotational speed parameters, as well as the duration of the steps of the process, were adjusted to result in GNPs with a maximum lateral size in the range of 10 μm. 

PA12, or Nylon 12, is a nylon polymer with the formula [(CH_2_)_11_C(O)NH]_n_. It has a melting point of 178 °C, thermal conductivity of 0.3 W/(mK), and specific heat capacity of 1.7 J/(g·°C), while the linear CTE is 10^−6^/K and service temperature up to 100 °C. The electrical resistivity is about 2.0e^+15^ ohm·cm. In order to achieve accurate dosing of both polymers and fillers, PA12 polymer pellets (Rilsan AMNO TLD) were first ground into powders using a Retsch ZM200 lab-scale knife grinder equipped with a 500 µm sieve. A dry blend was then carried out at designed ratio by mixing the obtained powders and various reinforcement fillers: GNPs, CNTs, and CNT-modified chopped CFs. A masterbatch of highly concentrated polymer compound was manufactured by using a kneader-type extruder with the appropriate ratio of polymer and filler. The desired ratio of the filler was achieved by diluting the produced masterbatch with the base polymer on a twin-screw extruder, leading to higher shear forces and better filler dispersion. Two-millimeter thick plates were manufactured using thermal compression under press at 250 °C for the evaluation of the thermal conductivity of this composite material. 

### 2.2. Characterizations

A JEOL 7000 FE SEM and a Hitachi TM3030Plus SEM were used to characterize the enforcements of nano-inclusions/CFs and the composite. A Nikon XT H225 was used to scan the composite samples to carry out 3D characterization. The versatile XT H 225 systems offer a 225 kV microfocus X-ray source with 3 µm focal spot size. XT Software brings the fastest reconstruction of CT data with processing software, such as VolumeGraphics. It can be used for the inspection of internal faults of the plastic parts. The internal structural information of the samples was observed and collected by the compact desktop Bruker micro-CT, 3D X-ray scan system, SkyScan 1272 at NTUA. The system consists of a microfocus sealed X-ray source which operates at 20–100 kV and 10W, an X-ray detector with a maximum resolution of 11 Mp (4032 × 2688 pixels), and a 14-bit cooled CCD fiber optically coupled to a scintillator.

Raman spectra were measured using a micro-Raman spectrometer (Invia Reflex, Renishaw, Wotton-under-Edge, UK). An Ar+ laser operating at 514.5 nm was employed as the light source. The laser beam was focused to an approximately 1 µm spot on the fiber surface using a microscope objective of 100×. The power of the incident light to the sample surface was retained below 1 mW to avoid local overheating.

### 2.3. Theremal Conductivity and Electrical Conductivity Test Methods

The laser flash method was used to measure thermal diffusivity. A laser flash delivers a short pulse of heat to the front of the sample, and an infrared scanner observes the temperature change at the rear face as a function of time. With a reference sample, specific heat can be determined (C_p_), and with measured density (ρ) and thermal diffusivity (α), the thermal conductivity (K) at a certain temperature can be calculated with the following equation:

K = α·C_p_·ρ (1)

where K is the thermal conductivity (W·m^−1^·K^−1^), α is the thermal diffusivity (m^2^/s), C_p_ is the specific heat capacity (J·Kg^−1^·K^−1^), and ρ is the density of the sample (kg/m^3^). A NETZSCH thermal analyser DSC 404C was used to measure the specific heat capacity. A NETZSCH LFA 427 with a TASC 414/4 controller was used to measure the thermal diffusivity.

Electrical conductivity measurements were made using a Keithley 6517B electrometer in the 2-point measurement mode. Five-centimeter long compound extrudates (small rods) were covered on the edges using silver paste, and their diameter was measured. Resistance was measured under 1, 10, or 100 V depending on the compound conductivity. Volume conductivity σ is calculated as follows:

σ = L/(R × S)(2)

where L is the sample length in cm, R is the measured resistance in ohms, and S is the rod section in cm². Conductivity values are given in S/cm².

### 2.4. FFF Process Parameters

**Figure 1:**
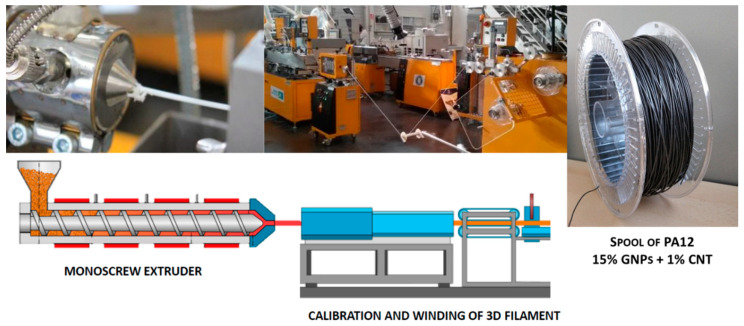
3D printing fillers produced by a single-screw extruder combined with a filament shaper.

3D printing filament manufacturing was performed at 230 °C using a single-screw extruder with a water-cooling bath combined with a filament shaper to calibrate a diameter of 1.75 mm and 4 km long. The extrusion process and the produced spool of PA12_15%GNPs + 1%CNTs can be seen in Figure 1. The filament was evaluated by TGA and DSC, and the technical detail is listed in Table 1.

**Figure 2:**
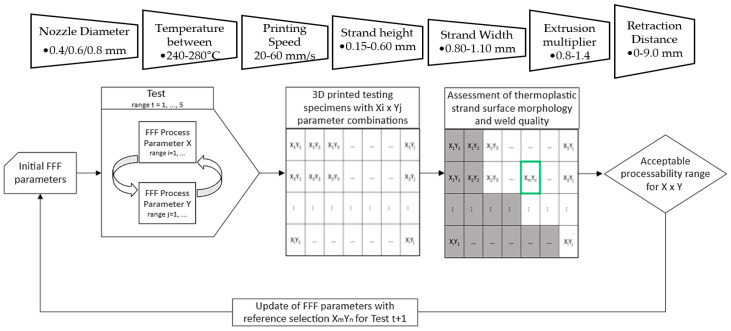
FFF Processability optimization path.

For FFF processability assessment, filament segments of 30 cm were cut and measured with a digital caliper to determine the average diameter of each segment. Filament drying was conducted by a hot-air drying oven at 80 °C for 12 h, and all segments were conditioned prior to testing in a low-humidity enclosure. The Raise 3D Pro 2 Plus FFF system was employed for the fabrication of testing specimens with different process parameters, and the generation of custom Gcode scripts was conducted through 3DOptimizer software (FabControl SIA). Firstly, a preliminary extrudability assessment was conducted for three nozzle diameters (0.4, 0.6, 0.8 mm) in order to assess flow consistency and extrusion temperature range. After the nozzle diameter (0.8 mm) was selected, seven different temperatures above the melting temperature (189 °C in Table 1) were tested, from 240 °C to 280 °C, for printing quality examination. Subsequently, sequential interdependence tests were conducted by varying nozzle temperature, printing speed, extrusion multiplier (volumetric flow rate compensation factor), retraction, and thermoplastic strand width and height as seen in Figure 2. Precise leveling and calibration of the FFF system and first layer settings were conducted prior to each test. Stereomicroscope evaluation (S9 stereomicroscope, Leica Microsystems) was conducted to assess morphology and deposition consistency of thermoplastic strands, aiming to identify parameter sets that eliminated structural defects related to over/under-extrusion and presented high strand uniformity. 

## 3. Results

### 3.1. Carbon-Based Nanofillers

**Figure 3:**
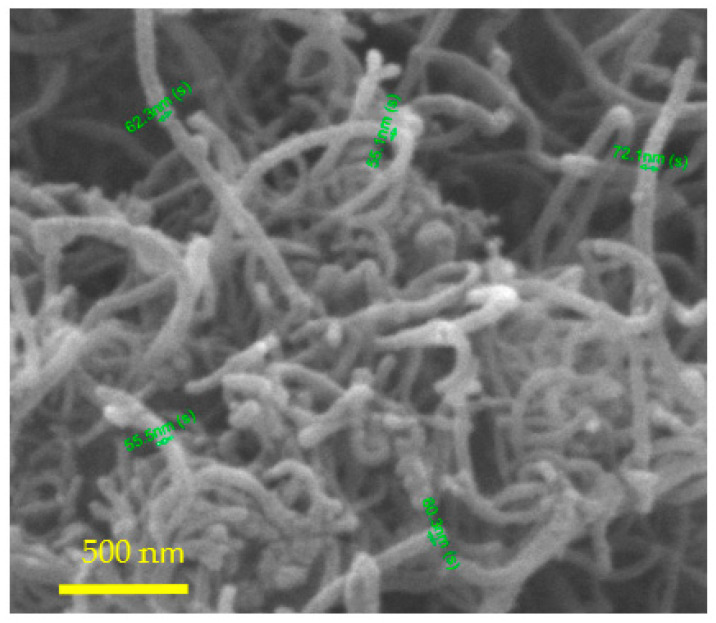
SEM images of MWCNTs prepared with the supported catalyst approach (Catalyst: Fe/zeolite; carbon source: acetylene).

Multiwalled carbon nanotubes (MWCNTs) were prepared with the supported catalyst approach and had an average diameter of 50–80 nm, as shown in Figure 3. They were in an entangled form, with embedded catalyst at their end caps, implying a tip growth mechanism.

**Figure 4:**
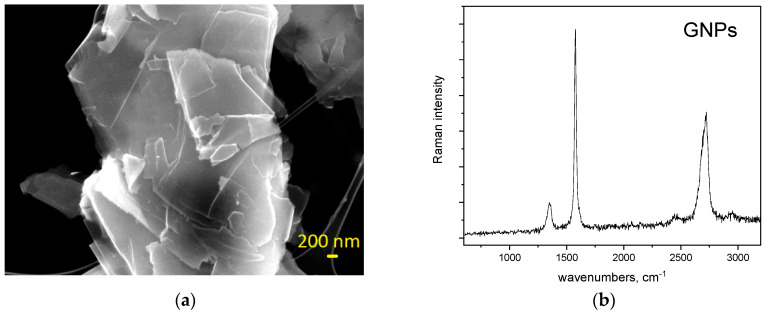
(**a**) SEM images of GNPs and (**b**) Raman spectra of the GNPs.

The GNPs powders were dispersed on adhesive conductive tape for SEM characterization. The platelets had a lateral dimension within the range of 2–10 μm, with thickness of a few tens of nanometers. The material was partially exfoliated (or reaggregated), with platelets showing highly exfoliated regions together with thicker crystals (Figure 4a). The GNPs Raman spectra present very sharp and high-intensity G (1580 cm^−1^) and 2D (2680 cm^−1^) lines, which corresponds to high-crystallinity and low-defects surface structure (Figure 4b). The most prominent “disorder” Raman spectrum, D-line (1360 cm^−1^), suggests an increase in the amount of crystallite boundaries with decreasing crystal size or to the presence of graphite edge planes perturbing the smoothly stratified crystallites. 

**Figure 5:**
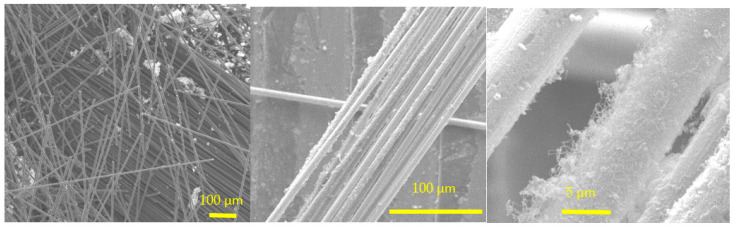
CNTs on chopped CFs (floating catalyst approach: catalyst: ferrocene; carbon source: ethanol).

Different procedures were tested for the growth of CNTs on chopped CF surface. As displayed in Figure 5, the CFs grafted by the floating catalyst approach were homogeneously coated with thin CNTs, surrounding each monofilament.

### 3.2. Composite Structure Characterization by SEM/Micro-CT

**Figure 6:**
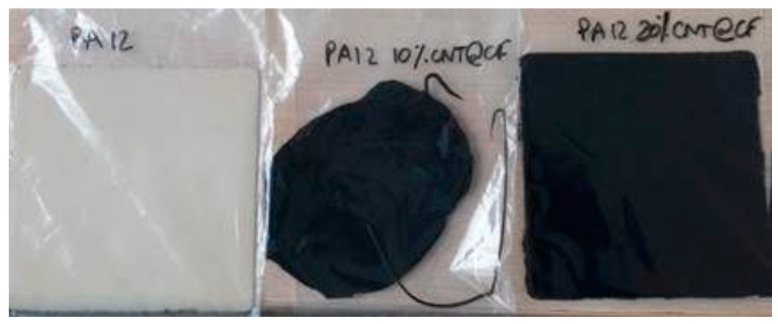
The fabricated coupons of PA12 and PA12 with 10% and 20% inclusions of CNTs@chopped CFs.

**Figure 7:**
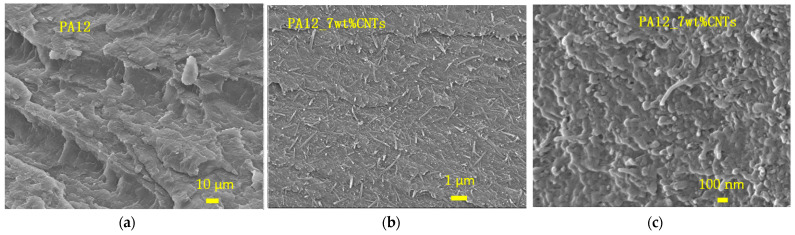

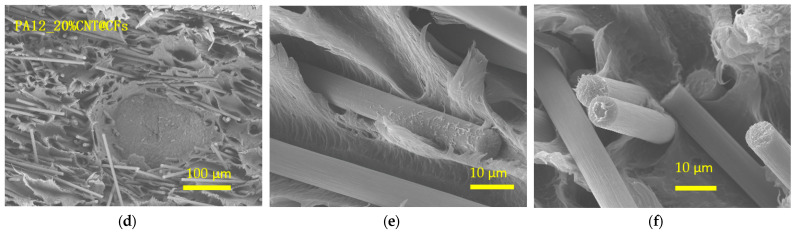
SEM fractography of (**a**) the neat PA12, (**b**,**c**) PA12 with 7 wt% CNTs, and (**d**–**f**) the PA12 with 20 wt% CNTs@CFs.

PA12-based polymer composites with different functional additions (CNTs, CNTs@chopped CFs, and GNPs + CNTs) were made to enhance their thermal conductivity (Figure 6). The fractography of the PA12 neat composite and CNT-modified PA12 was observed by SEM, as shown in Figure 7a–c. The CNTs are evenly distributed in the PA12 composite, and no pore or agglomeration can be seen (Figure 7b,c). Although most of the chopped CFs are evenly distributed in the PA12 matrix, some space can be seen at the interface of the CFs and the resin as displayed in Figure 7d–f. Pores and agglomerations can also be seen in the matrix of 20wt%CNTs@CFs inclusion. 

**Figure 8:**
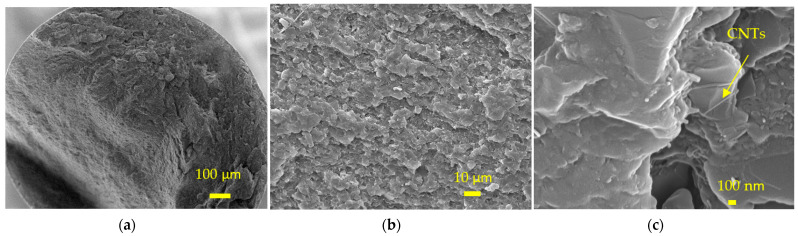
SEM fractography of the PA12-15wt%GNPs+1wt% CNT sample (**a**) the fracture, (**b**) an enlarged area, and (**c**) the CNTs in the composite.

A layered structure can be seen on the fractography of the PA12_15%GNPs+1wt%CNT sample (Figure 8). The composite has a dense structure, and the CNTs are well embedded in the composite (Figure 8c).

**Figure 9:**
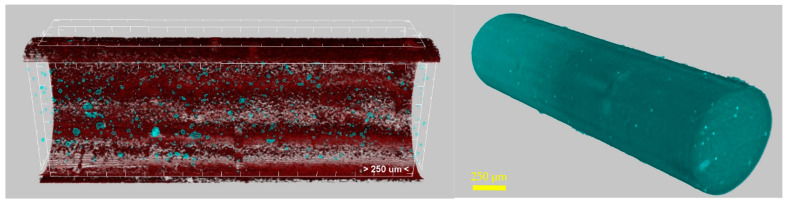
Micro-CT analysis images of PA12_15% GNPs + 1%CNTs.

The macro-scale observations regarding the surface quality of the filament are also confirmed through the micro-CT analysis (Figure 9 and Table 2). The shape of the filament appears to be circular and is within acceptable limits, as most areas are quite dense but some macrocracks can be seen in the pellet. Some defects and irregularities on the surface can be attributed to possible agglomerates that form during the manufacturing of the filament. 

**Figure 10:**
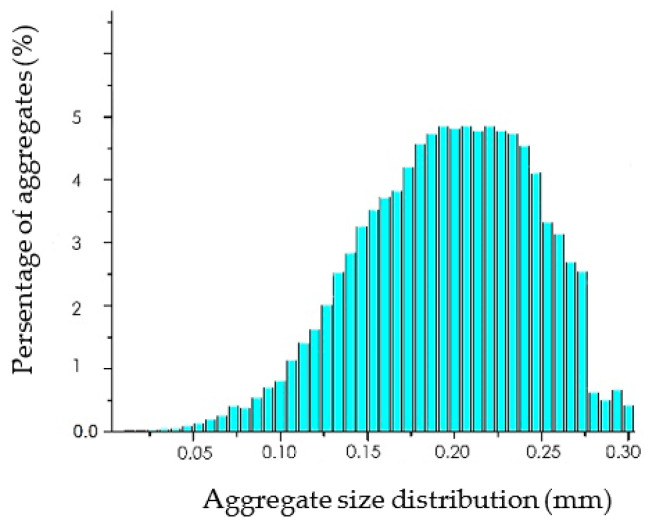
Aggregate size distribution for PA nanocomposite filament.

Nanomaterial aggregation in the composite filament was also investigated, and its influence on the final products’ properties was determined through CTAn software. The PA nanocomposite filament’s aggregates constitute 1.2% in volume, with mid-range aggregation sizes placed at 0.18–0.25 mm (Figure 10). For this measurement, the similarity in structure between the organic polymer matrix and the carbon-based additives causes similar X-ray absorption between the two. From the 3D visualization and the indicative reconstructed slices (Figure 9 and Figure 10), it is illustrated that the PA12 nanocomposite filament exhibits needle-like aggregations as well as clusters of aggregations near the filament surface, which may also cause surface morphological variations. Such defects increase the possibility of printing inconsistencies in the final product fabrication process, and, therefore, printing parameters (e.g., nozzle diameter) should be modified. 

### 3.3. Thermal Conductivity and Electrical Conductivity Results

**Figure 11:**
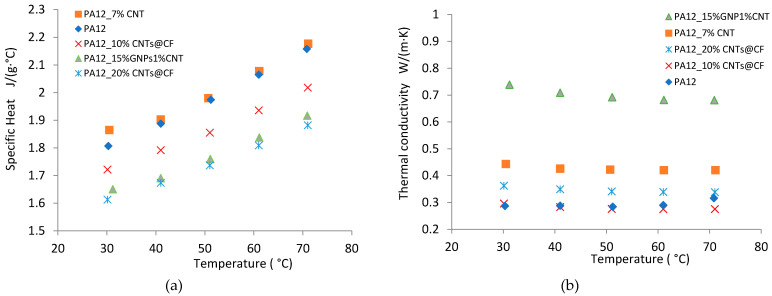
Thermal conductivity of PA12 incorporated with different reinforcements: (**a**) specific heat and (**b**) calculated thermal conductivity.

The density (Table 3), the specific heat, and the thermal diffusivity of the PA12-based composite were measured, and the thermal conductivities were calculated and plotted in Figure 11. In comparison with the neat PA12, 20 wt%CNTs@CFs inclusion increased the thermal conductivity of PA12 slightly, but 10 wt% CNTs @ CFs inclusion showed no improvement, especially at elevated temperature (Table 3 and Figure 11). Micro-CT scan and SEM examination indicated that there were many pores and agglomerations in the composite (Figure 7d–f), which resulted in less effective thermal conductivity enhancement. With 7 wt% CNTs inclusion, the thermal conductivity of PA12 increased significantly at the test temperature range of 30/40/50/60/70 °C. The CNTs were evenly distributed in the PA12 (Figure 7b), which led to a significant improvement in thermal conductivity. Furthermore, sample PA12_15GNPs + 1% CNTs performed best and nearly tripled the thermal conductivity value of the neat PA12 at the test range thanks to the inclusions of GNPs and CNTs (Figure 8). 

The measured electrical conductivity of neat PA12 compound was 1.00 E^−12^ S/m, and it increased to 1.00 E^−10^ S/m for the 10 wt% CNTs @ CFs-modified PA12, while the value reached 6.00 E^−6^ S/m for the 15 wt% GNPs + 1 wt% CNTs-modified PA12 compound. 

### 3.4. FFF Processability Assessment

Due to its improved thermal conductivity and low electrical conductivity, PA12 compound with 15 wt% of GNPs and 1 wt% CNTs was used for the FFF processability assessment. A range of process parameters were investigated for FFF processing, as shown in Figure 2 and Table 4. The upper and lower limits of each parameter value have been set from preliminary tests to ensure consistent flow through the nozzle and according to the FFF system operational range.

**Figure 12:**
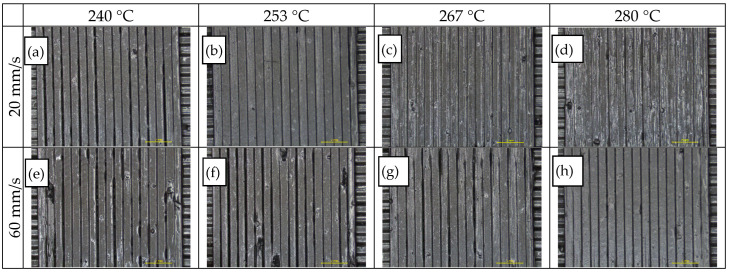
Optical inspection via stereomicroscope (7×) of specimens obtained from varying extrusion temperatures (ET) and printing speeds (PS): (**a**–**d**) SP = 20 mm/s and ET = 240, 253, 267, 280 °C from left to right; (**e**–**h**) SP = 60 mm/s and ET = 240, 253, 267, 280 °C from left to right.

A total of 27 variants with seven extrusion temperatures and four different printing speeds were studied to find the effect of extrusion temperature and printing speed. As both parameters directly affect the maximum volumetric flow rate through the nozzle, the optimum range is obtained once printing speed is in balance with the maximum material throughput, while over- and under-extrusion is observed when this balance is disrupted. As shown in Figure 12a–h, for the upper (60 mm/s) and the lower (20 mm/s) printing speeds, the impact of the printing temperature increases, as demonstrated through a transition from gap formation to over-extrusion due to the associated variation in material melt viscosity. By inspection of thermoplastic strand surface morphology and weld quality among adjacent strands, the acceptable limits for printing temperature and speed were 253–280 °C and 20–47 mm/s respectively, while the combination of 280 °C and 33 mm/s was selected as reference for further assessment.

**Figure 13:**
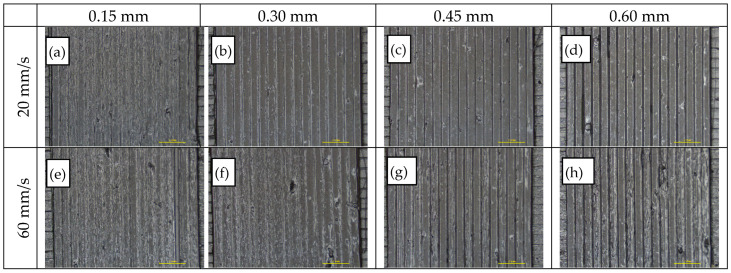
Optical inspection via stereomicroscope (7×) of specimens obtained from varying strand heights (SH) and printing speeds (PS): (**a**–**d**) SP = 20 mm/s and SH = 0.15, 0.30, 0.45, 0.60 mm from left to right; (**e**–**h**) SP = 60 mm/s and SH = 0.15, 0.30, 0.45, 0.60 mm from left to right.

For the selected extrusion temperature, the effect of strand height was assessed for the same range of printing speeds by combining seven strand height values with four printing speed values investigated in the previous step, in a total of 27 variants. As the strand height value (equal to layer height) is employed for the automatic calculation of the filament feed rate, a variation of this value can lead to over- or under-extrusion when the nominal volumetric flow rate is not in accordance with the actual volumetric flow rate required to achieve the targeted strand volume. As shown in Figure 13a–h, for the two upper and lower limit values of printing speed, the effect of nominal strand height increase is demonstrated through a transition from increased overlapping of adjacent strands and rough surface morphology associated with over-extrusion to gap formation due to the associated deviation between nominal/actual volumetric flow rate. Acceptable limits for strand height and printing speed were defined as 0.30–0.45 mm and 20–47 mm/s, respectively. Furthermore, seven strand widths were studied at a printing speed of 33 mm/s and strand height of 0.3 mm, and it was found that the acceptable limits for strand width were between 1.00 and 1.10 mm.

**Figure 14:**
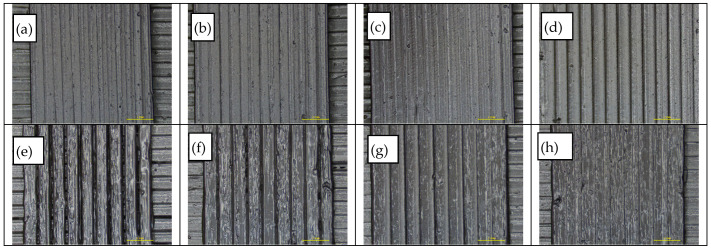
Optical inspection via stereomicroscope (7×) of specimens obtained from varying strand widths (SW) and the extrusion multiplier (EM): (**a**–**d**) SW = 0.80, 0.90, 1.00, 1.10 mm from left to right; (**e**–**h**) EM = 0.8, 1.0, 1.2, 1.4 from left to right.

For parameters selected in previous steps, the effect of strand width was assessed for seven strand width values, employed (along with the strand height) for the automatic calculation of the filament feed rate speed. As displayed in Figure 14a–d, for the selected printing speed of 33 mm/s and strand height of 0.3 mm, the effect of strand width increase is similarly demonstrated through a transition from increased overlapping of adjacent strands to uniform strand width deposition close to the nominal value. Acceptable limits for strand width were defined between 1.00 and 1.10 mm, while the value of 1.10 mm was selected as reference for further assessment.

In the next step, for parameters selected previously, the effect of a volumetric flow rate compensation factor (extrusion multiplier) was investigated for seven extrusion multiplier values. The extrusion multiplier allows for an automatic percentage adjustment of the nominal volumetric flow rate value by multiplying by a user-defined value. As depicted in Figure 14e–h, the effect of extrusion multiplier adjustment can be employed for further fine tuning of strand geometry and intra-strand coalescence. Acceptable limits for the extrusion multiplier were defined between 1.0 and 1.2, while the value of 1.2 was selected as reference for further assessment.

**Figure 15:**
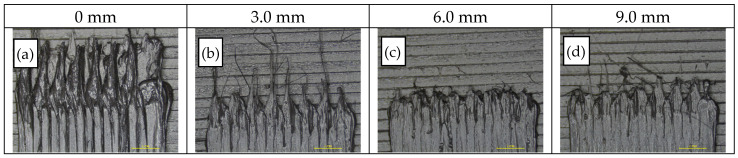
Optical inspection via stereomicroscope (7×) of specimens obtained from varying retraction distances (RD): (**a**–**d**) RD = 0.0, 3.0, 6.0, 9.0 mm from left to right.

Finally, retraction distance was investigated for seven values, at the selected printing speed of 30 mm/s, as a parameter related to the length of filament (in mm) that is reverted and re-pushed through the FFF system feeding mechanism during the printing head non-extruding movements. Retraction distance is employed for the control of surface defects (e.g., blobs and strings) resulting from material remaining in the nozzle tip. As demonstrated in Figure 15a–d, by inspection of thermoplastic strand end points where defects are manifested, acceptable limits for retraction distance were defined between 4.5 and 7.5 mm, while further increases of the value did not present any further enhancement and some remaining defects were not eliminated. 

In short, the extrusion temperature, printing speed, strand height and width, extrusion multiplier, and retraction distance all have an impact on the processability of FFF, and a combination of the optimized processing parameters is highlighted in Table 4.

## 4. Conclusions

Carbon-based nanofillers, CNTs, CNTS @ chopped CFs, and GNPs/CNTs were used to modify PA12 to improve its thermal conductivity. CNTs @ chopped CFs can improve the thermal conductivity of PA12 slightly, but a large amount is needed. Moreover, 7 wt% CNTs improved the thermal conductivity and electrical conductivity of the PA12, but they also led to agglomeration. PA12 with 15 wt% of GNPs and 1 wt% CNTs demonstrated the most improved thermal conductivity, and a processability assessment for FFF 3D printing technology was conducted to determine the processing window for the selected composition. Stereomicroscope optical inspection was conducted to assess morphology and deposition consistency of thermoplastic strands for a series of sequential interdependence tests varying nozzle temperature, printing speed, extrusion multiplier (volumetric flow rate compensation factor), retraction, and thermoplastic strand width and height. For the selected range of printing speeds, the impact of printing temperature increase was demonstrated through a transition from gap formation to over-extrusion, while the strand geometry obtained from different nominal strand height and width values provided insight into the actual volumetric flow rate and acceptable limits of nominal values to obtain strand uniformity. Finally, the effect of a volumetric flow rate compensation factor was investigated to further optimize inter-strand coalescence and filament retraction during printing head non-extruding movements; this indicated a moderate control of surface defects (e.g., blobs and strings) resulting from material remaining in the nozzle tip. Based on the results obtained, parameter sets were defined to achieve high strand uniformity and coalescence for the nanocomposite filament, eliminating most structural defects related to over-/under-extrusion and, thus, meeting the necessary criteria to be processable with FFF technology.

## Figures and Tables

**Table 1:** Technical data of the PA12_15%GNPs + 1%CNTs filament.

Base Polymer Material	PA12 AMNO TLD
Fillers	15 wt% GNP + 1 wt% CNTs
Melting point (°C)-peak	189 °C
Glass transition temperature (°C)	51 °C
Max. printing temperature (°C)	280 °C
Diameter	1.75 mm
Color	Black
Translucency	No
Density (g/cm^3^)	1.16
Humidity take-up (w%)	~0.35%

**Table 2:** Micro-CT arithmetical results for the filament.

Parameters	Filament
Object Surface Density (mm^−1^)	3.82
Structure Thickness (mm)	0.58
Structure Separation (mm)	0.05
Structure Linear Density (mm^-1^)	1.72
Degree of Anisotropy	5.6
Fractal Dimension	2.09
Total Porosity (%)	0.18

**Table 3:** Summary of PA12 compounds modified by different filaments.

Sample	Detail	Density (g/cm^3^)	Thermal Conductivity at 30 °C (W/mk)	Thermal Conductivity Improvement vs. Neat PA12
PA12	Neat	1.005	0.28	-
PA12_7% CNT	7 wt% CNTs	1.124	0.44	57%
PA12_15%GNP1%CNT	15 wt% GNPs + 1 wt% CNTs	1.065	0.73	161%
PA12_10%CNT@CFs	10 wt% CNTs @ CFs	0.988	0.3	7%
PA12_20%CNT@CFs	20 wt% CNTs @ CFs	1.117	0.37	32%

**Table 4:** List of 3D printing parameters in the FFF processability assessment for PA12 compound with 15 wt% of GNPs and 1 wt% CNTs.

Extrusion temperature (°C)	240	247	253	260	267	273	**280**
Printing speed (mm/s)	20	**33**	47	60	-	-	-
Strand height (mm)	0.15	0.22	**0.30**	0.37	0.45	0.52	0.60
Strand width (mm)	0.80	0.85	0.90	0.95	1.00	1.05	**1.10**
Extrusion multiplier	0.8	0.9	1.0	1.1	**1.2**	1.3	1.4
Retraction distance (mm)	0	1.5	3.0	4.5	**6.0**	7.5	9.0

## Data Availability

Data are contained within this article.
